# Using administrative health data for palliative and end of life care research in Ireland: potential and challenges

**DOI:** 10.12688/hrbopenres.13215.2

**Published:** 2021-05-26

**Authors:** Maria Kelly, Katie M O'Brien, Ailish Hannigan

**Affiliations:** 1 National Cancer Registry Ireland, Building 6800, Cork Airport Business Park Kinsale Road, Cork, T12 CDF7, Ireland; 2 School of Medicine, University of Limerick, Limerick, V94 T9PX, Ireland; 3 Department of Health, Block 1 Miesian Plaza, 50 - 58 Lower Baggot Street, Dublin, D02 XW14, Ireland; 4 Health Research Institute, University of Limerick, Limerick, V94 T9PX, Ireland

**Keywords:** administrative health data, data linkage, palliative and end-of-life care

## Abstract

**Background**: This study aims to examine the potential of currently available administrative health and social care data for palliative and end-of-life care (PEoLC) research in Ireland. Objectives include to i) identify data sources for PEoLC research ii) describe the challenges and opportunities of using these and iii) evaluate the impact of recent health system reforms and changes to data protection laws.

**Methods**: The 2017 Health Information and Quality Authority catalogue of health and social care datasets was cross-referenced with a recognised list of diseases with associated palliative care needs. Criteria to assess the datasets included population coverage, data collected, data dictionary and data model availability, and mechanisms for data access.

**Results**: Nine datasets with potential for PEoLC research were identified, including death certificate data, hospital episode data, pharmacy claims data,  one national survey, four disease registries (cancer, cystic fibrosis, motor neurone and interstitial lung disease) and a national renal transplant registry.  The *ad hoc* development of the health system in Ireland has resulted in i) a fragmented information infrastructure resulting in gaps in data collections particularly in the primary and community care sector where much palliative care is delivered, ii) ill-defined data governance arrangements across service providers, many of whom are not part of the publically funded health service and iii) systemic and temporal issues that affect data quality. Initiatives to improve data collections include introduction of i) patient unique identifiers, ii) health entity identifiers and iii) integration of the Eircode postcodes. Recently enacted general data protection and health research regulations will clarify legal and ethical requirements for data use.

**Conclusions**: Ongoing reform initiatives and recent changes to data privacy laws combined with detailed knowledge of the datasets, appropriate permissions, and good study design will facilitate future use of administrative health and social care data for PEoLC research in Ireland.

## Background

Administrative health data is generated through the provision and administration of health and social care by health care service providers and other institutions.

Its use for research purposes is an area of growing interest ^1– 5^. Internationally initiatives to harness the potential of linked administrative health data for research purposes are well developed in Australia ^6– 8^, the UK ^9– 11^ the Nordic countries (Norway, Sweden, Finland, Denmark, Greenland and Iceland) ^12^ and Canada ^13^. However data linkage is a complex process and methodologies vary ^14, 15^. In Ireland the need to develop a coherent and integrated approach to health information ^16^ and the potential of routine data for health research ^17^ is recognised. Initiatives to harness that data for research purposes have started but these are at an early stage ^18^. The Health Service Executive (HSE), Ireland’s public health and social care service provider, is developing an open data strategy in recognition of the fact that the data it holds are a valuable asset that can improve healthcare delivery and planning ^19^.

Kane *et al.* reported that 80% of deaths in Ireland between 2007–2011 were from conditions recognized as having associated palliative care needs, with 30% of deaths from cancer and 50% from non-cancer conditions including neurodegenerative disease and dementia ^20^. Significant barriers to high quality palliative care research exist ^21– 23^. These include identifying and recruiting subjects, increased ethical concerns for vulnerable patients who are often seriously ill and methodological concerns including loss to follow up, recall bias or difficulties measuring endpoints such as pain or symptom burden ^22^. Some of these issues can be addressed using routine data. Davies *et al.* described a number of initiatives that use routine data for palliative and end of life care (PEoLC) research in England and elsewhere ^2^. The study identified three priorities for the future use of routine data; these were i) safe and ethical access to data, ii) improved data linkage and iii) improved PEoLC data collections. In Belgium, Maetens *et al.* identified and described the steps to access, interrogate and link seven population level databases for end-of-life research ^24^. In Ontario, Tanuseputro *et al.* used a range of routine data sources to examine the delivery of palliative care across acute care, outpatient clinics, and home care health sectors at the population level ^25^.

In Belgium, health insurance is legally mandatory so that data discovery relied mostly on access to claims databases which are managed by a single agency. In Ontario, claims data from Ontario Health Insurance Plan database augmented by linkage to a number of other administrative databases held at a single institute, formed the basis of data capture. These studies demonstrate that while there are universal challenges to using administrative health data for research, the context is local and requires examination at the local level. Initiatives to use administrative health and social care data in Ireland are beginning but to-date its use for quantitative PEoLC research ^26, 27^ has been limited.

Our aim is to identify the challenges and opportunities of administrative health and social care data for PEoLC research in Ireland. The study is timely given the recent initiatives to realise the potential of Irish health data ^18, 19^ and the emerging body of international studies using administrative health data ^2, 24, 25^. Our objectives are i) to identify administrative health and social care data available that may be useful for PEoLC research ii) to describe both the challenges and opportunities using these data for PEoLC based on our experiences to-date using linked cancer registry data, hospital episode data and death certificate data and iii) to describe how recent initiatives to improve the health information environment and changes to data protection laws will impact future use of administrative health and social care data in Ireland.

## Methods

### Setting

Ireland has a mixed public private health care system where publically funded health care is managed by the HSE and funded through the tax system. All residents are entitled to use the public health system. There are three private health insurance providers in Ireland and in 2018, 45% of the population had private health insurance ^28^. Privately insured patients in Ireland may be treated in public or private hospitals. The HSE National Clinical Programme for Palliative Care oversees the management and organisation of palliative care services in Ireland ^29^. Specialist palliative care is delivered by the HSE along with a number of voluntary service providers. Specialist palliative care teams provide care in acute hospitals, community settings and specialist inpatient units across the country.

### Identifying potential datasets for PEoLC research

The Health Information and Quality Authority (HIQA) is an independent body that evaluates the quality of the information available on health and social care and makes recommendations to improve quality, minimise inconsistencies and fill gaps where data are not available ^30, 31^. Quality is defined as data that are complete, valid, accurate, reliable, relevant, legible and available in a timely manner ^32^. HIQA advocates eight guiding principles for organisations collecting data that include formalised governance arrangements, facilitating appropriate access to the data to optimise its benefits, continuous monitoring/improvements of data quality and effective information governance procedures. Standards for data quality include the use of data dictionaries, classification systems and clinical terminologies ^30^. A data dictionary is a descriptive list of names, definitions and attributes of data elements to be collected in an information system or database and aids in the standardisation of data definitions ^33^. Related to the concept of data dictionaries, data models describe how the data are organised and stored within an information system or database. This affects how relevant data from different systems data can be identified, extracted and compared. Data dictionaries and data models hold data about the data, also called metadata.

HIQA produces a catalogue of national health and social care data collections using a standardised template to describe existing data collections ^16^. The 2017 catalogue was cross-referenced with a recognised list of diseases with associated palliative care needs ( Table 1), based on a methodology by Murtagh *et al.* ^34^. Given Ireland’s aging population and identified future palliative care needs for cancer, neurodegenerative disease and dementia ^20^ particular focus was given to disease registry collections. Criteria used to assess the datasets included an examination of population coverage, the data collected, the availability of data dictionaries and data models and information on how the data can be accessed.

**Table 1.** Conditions associated with palliative care needs and their International Classification of Disease codes.

Condition	ICD-10 codes *
Malignant neoplasm	C00-C97
Heart disease, including cerebrovascular disease	I00-I52, I60-I69
Renal disease	N17, N18, N28, I12, I13
Liver disease	K70-K77
Respiratory disease,	J06-J18, J20-J22, J40-J47 & J96
Neurodegenerative disease	G10, G20, G35, G122, G903, G231
Alzheimer’s, dementia and senility	F01, F03, G30, R54
HIV/AIDS	B20-B24

Source: 34, * 40

## Results

In total, nine datasets were identified from the HIQA catalogue with potential for PEoLC. These include population based death certificate data, hospital based episode data for all patients treated in public acute hospitals in Ireland, pharmacy claims data for all people eligible for medical cards, one nationally representative cohort study of people aged 50 and over, four disease registry collections and a national renal transplant registry. Four have data dictionaries and six have a process to request access to the data. To our knowledge there is no record of a requirement for payment (for non-commercial organisations) to access any of the data sources mentioned here ^35^. Key characteristics of the datasets are described in Table 2. Based on our previous experience using cancer registry data linked to death certificate data and hospital episode data ^36, 37^ we describe the strengths and weaknesses of these datasets for PEoLC research.

**Table 2.** Health and social care datasets with national coverage potentially relevant for palliative and end-of-life care (PEoLC) research.

Name	Disease	Coverage	Brief description	Data level	Data dictionary	Minimum dataset	More information
Vital Statistics - Death Registration	All	National population based	Death certificates	Individual	No	No	Further information on data for research can be found at https://www.cso.ie/en/aboutus/lgdp/csodatapolicies/ dataforresearchers/
Hospital In-Patient Enquiry(HIPE)	All	National for all acute public hospitals	A HIPE discharge record is created when a patient is discharged from (or dies in) hospital. Administrative, demographic and clinical information are collected for a discrete episode of care in 53 hospitals	Episode of care	Yes	Same as data dictionary	Datasets for HIPE discharges are provided to a number of State agencies in order to address specific data requirements. Data requests can be submitted by email to HIPEData.Requests@hpo.ie Further information on HIPE can be found at http:// www.hpo.ie/
Primary Care Reimbursement Service (PCRS).	All	Nationally to all population entitled to a medical card The data covers the main national health schemes throughout the country.	The PCRS is responsible for making payments to Healthcare Professionals, e.g. GPs, dentists, pharmacists and optometrists/ ophthalmologists, who provide free or reduced cost services to members of the public across a range of community health schemes.	Script based transactions	No	No	Reports based on PCRS data are available from the eHealth Ireland Open Data Portal at https://www. sspcrs.ie/portal/annual-reporting For more information see https://www.hse.ie/eng/ staff/pcrs/
The Irish Longitudinal Study on Ageing -TILDA	Not applicable	A nationally representative sample of adults aged 50 and over, resident in Ireland n=8,504	TILDA collects information on all aspects of health, economic and social circumstances from people aged 50 and over in a series of data collection waves once every two years beginning in October 2009.	Individual	Yes	Yes	Information on accessing the datasets are available from https://tilda.tcd.ie/data/accessing-data/ Further documentation is available at https://tilda. tcd.ie/data/documentation/
National Cancer Registry Ireland database (NCRI)	Malignant neoplasm	National population based	All incident tumours recorded	Individual	Yes	Yes	Anonymised aggregated data are available for download from the NCRI website. Requests for individual level data are examined on a case by case basis. See website for details https://www.ncri.ie/
Irish Motor Neurone Disease Register	Motor Neurone Disease	All known patients diagnosed with MND in the Republic of Ireland.	Hospital In-Patient Enquiry (HIPE) discharge data in all major hospitals are searched to ascertain and confirm all MND diagnosis. The Central Statistics Office (CSO) Deaths Register is searched to capture MND cases where the subject passed away shortly after diagnosis. The register is based on direct nationwide chart review/confirmation by the diagnosing physician.	Individual	No	No	Requests for data are considered on a case by case basis. Email: ResearchMND@tcd.ie Further information can be found at http://mnd.ie
Cystic Fibrosis Registry of Ireland (CFRI).	Cystic Fibrosis	All consenting persons with CF in the Republic of Ireland.	Participation is voluntary, enrolment is based on patient consent to have their medical record details added to the registry. Data is taken from patient medical charts by registry staff	Individual	On request	No	Further information and requests for registry data can be made by downloading and completing the CFRI Data Application Form
Irish Thoracic Society Interstitial Lung Disease Registry	Lung diseases including Interstitial Lung Disease (ILD)	All consenting persons with ILD in the Republic of Ireland	Participation is by consent. The registry aims to identify, record, analyse, and store information relating to the prevalence, incidence, and treatment of ILD in the Republic of Ireland.	Individual	No	No	Further information available by contacting the Irish Thoracic Society at https://irishthoracicsociety.com/contact-us/ and the ILD website at https://irishthoracicsociety. com/lung-disease/interstitial-lung-diseases/
National Renal Transplant Registry	Recipients of renal and pancreas transplants	All consecutive renal transplants from 1964 to date	The registry is maintained for the recipients of renal and pancreas transplants	Individual level	No	No	For further information contact Beaumont Hospital Kidney Centre. http://www.beaumont. ie/kidneycentre-home

### Death certificate data

Every death in Ireland is legally required to be notified to the state within three months of death, so death certificate data is population based at the national level. Death is a unique event so a person should only have one death certificate record. The Department of Social Protection, Central Statistics Office (CSO) and General Register Office collect and record date of death, address of residence of deceased, place of death, cause of death, occupation of deceased, age of deceased, sex of deceased, and marital status of deceased. Cause of death for all deaths registered from 2007 onwards are coded using ICD10 codes ^38^. Place of death is recorded as an address and is not classified e.g. into home, hospital, hospice, or long-term care facility ^39^. Information on how to access the data are available at https://www.cso.ie/en/aboutus/lgdp/csodatapolicies/dataforresearchers/.

### Hospital Episode data

The Hospital In-Patient Enquiry system (HIPE) collects demographic, clinical and administrative data on discharges from, and deaths in, all acute public hospitals nationally. Details of each episode of care is recorded as a single record so that over time an individual can have multiple records within and across HIPE hospitals. HIPE is the only source of morbidity data available nationally for acute hospital services ^41^. In 2016, 53 hospitals were contributing to HIPE. Data are not available in HIPE for emergency department attendances unless the patient is admitted to hospital. Data are also not available for 22 private hospitals. Clinical coders review the records of each patient and extract the relevant clinical data, and translate it into codes using the ICD-10-AM/ACHI/ACS 8th edition ^42^. As well as a source of clinical information for many chronic diseases with associated palliative care needs (e.g. dementia, neurodegenerative diseases and cancer), diagnostic codes include ‘Z51.5 - Palliative care’ recorded when a patient has been seen by the palliative care team ^42^. The guidance for recording palliative care in HIPE changed with the introduction of the 10 ^th^ edition ICD-10-AM/ACHI/ACS from January 2020. Palliative care should be recorded only where there is documented evidence that the patient has been provided with palliative care ^43^. Notwithstanding evidence of variation in how the code is used across hospitals, currently HIPE is the only available population level administrative dataset where a record of a patient being seen by a palliative care specialist can be identified ^44^. Additional relevant information for PEoLC research include admission type (elective/emergency) and patient destination on discharge with categories that include home, nursing home, transfer to another hospital, transfer to hospice and/or died. Information on accessing data and a data dictionary for HIPE data are available from http://www.hpo.ie/

***Opportunities for data validation.*** The Minimum Data Set (MDS) is a national survey of demographic and patient activity data for specialist palliative care services in Ireland ^45^. Monthly aggregate data from specialist palliative care inpatient units, community (homecare) services, day care services and acute hospitals are returned to a national office. The Specialist Palliative Care MDS does not contain patient level data and is not listed in the HIQA catalogue. A summary analysis of MDS ^45^ for the period 2012 to June 2016 reported several metrics including

- The number of new patients in receipt of inpatient specialist palliative care, community care, and day care.
- Place of care prior to admission to inpatient units.
- The number of admissions and discharges from inpatient units.
- Inpatient bed availability and occupancy.
- The provision of care to non-cancer patients.
- Wait times for inpatient care and community care.
- Specialist palliative care in the community and place of death.

Data from all acute hospitals was incomplete at the time of analysis and excluded from the report, however MDS aggregated data of specialist palliative care activity in acute hospitals in 2016 has been used to validate HIPE coding of palliative care ^37^.

### Primary care reimbursement service data

The HSE Primary Care Reimbursement Service (PCRS) is responsible for making payments to healthcare professionals including general practitioners (GPs), dentists and pharmacists, for the free or reduced costs services provided to the public under the General Medical Scheme and/or other schemes ^46^. Access to the schemes is means-tested on a rolling basis and/or determined by specified long-term disease. Qualifying individuals are given a medical card with a unique medical card number (MCN). Eligibility for a medical card can change with changing circumstances so that over time, one person can have had a number of medical cards. In 2018, 43.4% of the population (over 2 million people) were eligible for a medical or GP visit card ^47^. The PCRS dataset is one of the few national datasets that collects data in primary and community care settings. All expenditures around pharmaceuticals (drugs/medicine costs) are recorded against an MCN so that the data are transaction based. A data model is not currently available for PCRS so it is not clear how an individual is linked with medical card(s) within the PCRS database or whether an individual or a medical card is recorded more than once; neither is a data dictionary available. Further information on PCRS can be found at https://www.hse.ie/eng/staff/pcrs/.

### The Irish Longitudinal Study on Ageing

The first wave of data collection for the Irish Longitudinal Study on Ageing (TILDA) surveyed a nationally representative sample of over 8500 people, aged 50 years and over, beginning in October 2009 with a further four waves of data collection in 2012, 2014, 2016 and 2018 ^48^. Each individual within the TILDA dataset has a unique identifier and a wide range of data on the health, economic and social aspects of participants' lives are collected through personal interviews, self-completion questionnaires and health assessment measures ^49^. TILDA is unique in Ireland in that it contains detailed longitudinal data on education, income and occupation in this age group which is not readily available elsewhere.

TILDA data has been linked at the person level with death certificate data, matching on the basis of name, address and month and year of birth. Matching was performed for all individuals who died between Wave 1 (2009/2011) and March 2018, and of a total of 863 confirmed deaths among the TILDA sample, matching death records were obtained for 779 decedents (90.3%) ^50^. By law every death is registered and this is reflected in the high match rate achieved. Matching allows for detailed research around end of life care given the depth and breadth of information collected prospectively before death by TILDA. This work demonstrates person level data linkage with the other datasets described here is feasible. Anonymised data and documentation on TILDA are available for download from the Irish Social Science Data Archive ^51^.

### Cancer registry data

The NCRI collects data nationally for incident tumours recorded at the level of the patient, that is each patient should be recorded once only in the registry database. This relational data model simplifies data linkage where data are matched at the person level using demographic details. Over time a patient may have additional tumour and management data attached to their patient record. Information collected includes patient demographics (age and sex), type of cancer (site and staging), treatments and selected procedures, date and cause of death (from linked death certificate data). Clinical information is coded using international guidelines including international classification of diseases (ICD) codes ^40, 52^. The focus of data collection is on the first year post-diagnosis with limited data collection thereafter. Completeness of case ascertainment is estimated to be 98.7% ^53^. Information on accessing data from the NCRI is available from the website https://www.ncri.ie/.

### Other disease registries

Population based disease registries are a good starting point for PEoLC research because selection bias is reduced since the whole population with the disease are identified. The Irish Motor Neurone Disease (MND) register was established in 1995 and collects data on all known patients diagnosed with MND each year and currently it holds information on over 2,200 patients ^54^. Individual level demographic data are recorded so that linkage to HIPE, death certificate data and PCRS data should be feasible. Date of disease onset is also captured so that studies on the patient’s PEoLC needs throughout the disease trajectory are possible.

The Cystic Fibrosis registry requires patient consent for data collection. In 2017 it was estimated the registry coverage of the cystic fibrosis population was just over 90% ^55^. The characteristics of those patients not captured are unknown, so studies using the cystic fibrosis registry may be subject to selection bias. Detailed demographic information that includes name and address, date of birth and ethnicity are recorded. Additional information includes information on diagnostic tests, genotype, symptoms and method of diagnosis, age at diagnosis, number of hospitalisations between annual assessments, complications and other clinical data and social data. Data linkage to death certificate data, HIPE data and PCRS data should be feasible. The cystic fibrosis registry is unique among the datasets described here in that it collects ethnicity data ^56^.

The Irish Thoracic Society Interstitial Lung Disease (ITS-ILD) Registry began collecting data in 2016. Patient written consent is required for data collection. The first annual report (2018) on 154 patients found 46% of patients with idiopathic pulmonary fibrosis were referred through primary care, 12% of patients were referred for lung transplant assessment and 13% were referred to palliative care ^57^. A key finding is that most patients with idiopathic pulmonary fibrosis will need a lung transplant or palliative care. While only preliminary information on disease stage and no information on survival times were provided, the report demonstrates the value of disease registries in providing detailed information necessary to assess the need for palliative care services.

### National renal transplant registry

The National Kidney Transplant Service for the Republic of Ireland was established in 1986 and is coordinated through Beaumont Hospital ^58^. The National Renal Transplant Registry collects data on parameters at time of transplant, renal disease and source of transplant. Patient data collected includes gender, area of residence and date of birth. The information is used to assess graft survival and patient survival, monitor factors affecting outcome and monitor performance ^16^. Three further national transplant services exist in Ireland: the National Heart and Lung Transplant Service, the National Liver Transplant Service transplant and the National Pancreas Transplant Centre ^59^. Person level data from the transplant databases has been linked to cancer registry data to examine cancer incidence after organ transplantation ^60^.

## Considerations for PEoLC research

### Structural issues in healthcare organisation and delivery

The *ad hoc* development of the Irish health system has contributed to an information infrastructure that often does not link across service providers thus leading to duplication, fragmentation and increased costs. Patients cannot be easily tracked from hospital to community based care leading to large gaps and silos of under used data ^30^. Gaps exist particularly from the primary and community care sector as well as from outpatient clinics and emergency department attendances that don’t result in hospital admission. The lack of community and social care data is particularly relevant for PEoLC as a considerable amount of palliative care is delivered in the community.

### Data governance

A second consequence of the *ad hoc* development of services means how data is managed and accessed across providers, many of whom are not part of the HSE, is not well defined. Private hospitals do not contribute to the HIPE national data collection so that studies based on HIPE data cannot be generalised to the whole population. Biases and omissions in the available data cannot be adequately assessed. Similarly inpatient hospice services in Ireland are mostly provided by charities, partially funded by the HSE but with separate and distinct governance structures ^61^. Data models describing how the data are stored and organised are generally not available so that gauging the workload to manage and link data can be complex. Data dictionaries are generally not available so the datasets usefulness for PEoLC research cannot be evaluated in terms of the data items potentially available.

### Individual health identifiers

The 2014 Health Identifiers Act ^62^ mandated the creation of an individual health identifier (IHI) register so that all health service users can be uniquely identified. While work is ongoing to introduce IHIs across the Irish health system, they have not yet been widely incorporated into the national data collections described above. In the absence of unique identifiers, linking patient records across datasets requires probabilistic matching techniques ^63^, comprehensive strategies to guide the process including data cleaning and standardisation techniques ^64^ and detailed knowledge of the datasets to be linked. Address can be used in matching but over 35% of addresses in Ireland share their address with at least one other property. Eircode, Ireland’s postcode system, was launched in July 2015 where a unique postcode is assigned to each residential and business address. The integration of eircodes will facilitate probabilistic data matching of administrative datasets and allow geospatial analysis of the data.

Issues affecting quality or completeness of data within each dataset can affect the efficacy and accuracy of probabilistic matching. Issues can be systemic e.g. how the data are organised and stored. Temporal issues can include health service reconfigurations, changes to eligibility criteria (e.g. eligibility for medical cards) and/or changes in classifications systems over time.

### Health Service Providers Identifiers

The 2014 Health Identifiers Act ^62^ also legislated for the development of a national database to capture, maintain and publish quality assured and verified standard codes and identifiers for health related entities i.e. practitioners, organisations, services, locations, and information on the relationships between them ^65^. The repository will hold up-to-date information on health sites/locations, health care providers and services provided by the HSE and Private/Voluntary Organisations in Ireland. The introduction of health service provider identifiers will facilitate classification and enumeration of services that will benefit PEoLC research.

Place of death is an important outcome measure at a population level. In PEoLC research, place of death is commonly standardised to Own Residence, Hospital, Care Home and Hospice based on the place of death address, ^66^. There are no standards in use for Irish mortality data ^39^ so that categorising place of death based on the address of a healthcare facility can be difficult without local knowledge. Facilities range from specialised centres to large regional hospitals, general hospitals, community and district hospitals, public and private nursing homes. Some facilities provide different services on the same site e.g. nursing home and hospice services. Ambiguity around place of death could be reduced by requiring institutions to self-categorise the main services they provide from a standardised list.

### Electronic Health records

The introduction of a national Electronic Health Record (EHR) in conjunction with IHI’s are a key part of the HSE’s strategic e-Health Programme ^67^. EHRs are the means by which data can be recorded and shared across organisations and care settings ^68^. Core functions will allow electronic prescribing and case management as well as the ability to aggregate data from these systems into a comprehensive national record, accessible to health and social care professionals, patients, service users and carers.

The 2020 HSE National Service Plan commits to progressing procurement of an electronic health record (EHR) solution in the National Children’s Hospital which will inform the procurement of an EHR solution for all health and social care services [ 69, p. 20]. To-date an electronic health record had been introduced at several maternity hospitals in Ireland ^70^. Several projects benefitting from the improving electronic health infrastructure have already been realised ^71^.

 In Scotland the availability of electronic medical records have been used to develop electronic palliative care summaries to improve patient care for those accessing out–of-hours services ^72^. In England the impact of advance care planning (ACP) discussions have been evaluated in a hospice setting where that information has been recorded in the electronic patient record ^73^. A wider initiative that relies on the existence of electronic medical record has been in development for some time in England. The Electronic Palliative Care Coordination Systems aims to enable advance care planning, improve communication and coordination at end of life by providing up-to-date key information on patients believed to be in their last year of life ^74^. These studies from other countries demonstrate the opportunities for PEoLC research in Ireland as the electronic health infrastructure improves.

### Health region

Several reconfigurations of the Irish health service have occurred since 2005, each of which can impact the continuity and quality of data collected. For example health boards have been replaced by HSE administrative areas and more recently by Community Health Organisation areas (CHO). In 2019, the Sláintecare report recommendation for a ‘common unit of geography’ for data collection and integration to increase capacity for cross-organisational research (Information and Research, page 24) has been initiated with the announcement of six integrated health regions to replace the CHOs ^75^. The data collection systems have not kept pace with these changes so that a patient cannot be accurately assigned to a CHO area using address data alone. Eircode postcodes could be used to assign every household to a distinct CHO and/or other geographical units. This would eliminate any ambiguity for both service providers and service users on where to seek health care in the first instance, help establish criteria for access to services and facilitate meaningful research around service provision by health region. 

### General Data Protection Regulations

In May 2018 the General Data Protection Regulation (GDPR) became law in the European Union ^76, 77^. It regulates the processing of personal data relating to individuals in the EU so that personal data are

1. Processed lawfully, fairly and transparently.
2. Collected for specific legitimate purposes only.
3. Adequate, relevant and limited to what is necessary.
4. Accurate and kept up to date.
5. Stored only as long as is necessary.
6. Protected with appropriate security measures, ensuring its integrity and confidentiality.

Included in GDPR is the principle of patient consent where by valid consent from individuals is required for the processing of their personal data. Consent must be a “freely given, specific, informed and unambiguous indication of the individual’s wishes”. GDPR force a stricter data governance regime on organisations so that data controllers i.e. the organisations collecting data, can be required to prove compliance with GDPR requirements.

The measures for data processing for health research are given more specific effect through Ireland’s Health Research Regulations Act (HRR) ^78^. Some of the specific measures enshrined by HRR Act were considered restrictive ^79^. Of particular concern were the requirements for explicit consent or approval from the Health Research Consent Declaration Committee (HRCDC) ^80^ for ongoing research involving retrospective chart reviews, use of biobank materials and research with individuals who lack capacity to consent. In 2021 the Department of Health amended the HRR to address these issues and clarify situations where a health research declaration consent application is required ^81^.Under the new amendments, low risk retrospective chart reviews that have been approved by a research ethics committee and meet specified transparency requirements, no longer require a health research consent declaration ^82^.

The requirements for compliance with GDPR and in particular the HRR are complicated by the fragmented health data infrastructure. Guidance notes are available to assist data controller organisations when making an application to the HRCDC for a consent declaration. In addition a public log of HRCDC applications provide an insight to the working of the committee. Information on the decision process for existing applications that include the decision outcome, any specific conditions attached and/or additional recommendations can alert researchers to potential requirements and/or shortcomings in their own application ^80^.

## Discussion

Ireland does not have a universal healthcare system so there are no population-level insurance claims databases with national coverage unlike those used extensively in Belgium ^24^ and Ontario Canada ^25^ for PEoLC research. The Irish health system is characterised by a fragmented information infrastructure so that only death certificate data and a small number of the disease registry data collections are fully population based with national coverage. In this context, the use of cancer registry data complete with information on date, cause and place of death from linked death certificate data is a valuable tool for cancer PEoLC research. Because there is full coverage, biases due to missing data in the linked datasets can be better assessed and evaluated.

Studies using cancer registry data linked to hospital episode data for PEoLC research have been published in Ireland. One study examined the palliative care needs of lung cancer patients ^36^ and a second evaluated the receipt of specialist palliative cancer care in acute hospitals ^37^. A feature of these studies is careful study design driven by background knowledge of the data available. Cancer registry data allow cancer subtypes to be examined individually taking account of differences in survival, for example lung cancer is characterised by short survival times. For data protection reasons, only hospital episode data that mention a cancer diagnosis are made available to the cancer registry for linkage. Hospital episodes at or following cancer diagnosis and shortly before death, (particularly where cancer is a cause), are most likely to mention a cancer diagnosis and be provided to the cancer registry. In this context hospital episode data is more likely to be complete for cancers with short survival times, such as lung cancer.

Cancer registry data has also been linked to PCRS pharmacy claims data to examine the effects of drugs on cancer progression and survival ^83– 85^. In PEoLC, a goal of anticipatory prescribing is to allow patients have their symptoms managed at home at end of life ^86^. Anticipatory prescribing includes opioid for pain, sedatives for anxiety and agitation as well as anti-emetics for nausea and vomiting ^87^. In this respect the PCRS database could be a valuable resource for PEoLC research in the community where there is a recognised lack of data. Although not fully population based, a number of studies have described how PCRS can be used to study specific populations ^88^ and particularly those aged 70 years and over ^89^.

The Irish MND registry has been used extensively for research ^90– 92^, including an examination of the role of palliative care within a broader multidisciplinary approach to care ^93^. The evidence base for palliative care for neurodegenerative diseases in general is lacking for MND patients ^94^. A recent study has suggested certain triggers may be used to recognize the end-of-life phase in neurological patients. These include recurring infection, weight loss, dysphagia and aspiration pneumonia ^95^. Linkage to HIPE data to explore these triggers in MND patients may be one avenue for future research. 

In recent years lung transplantation for cystic fibrosis patients has become more common as patients survive longer with advances in care and treatment ^96^. The changing practices impact the location and intensity of end-of-life care of people with cystic fibrosis and warrants further examination ^97, 98^. The first report from the ITS-ILD Registry indicates most patients with IPF will ultimately need lung transplant or palliative care ^57^. For both the cystic fibrosis and the ITS-ILD registries, data linkage to administrative health data including HIPE data and death certificate data could be used to examine changing patterns in treatment and/or place of death.

Patients requiring transplant have advanced illness and may have unmet palliative care needs ^99– 101^. While specific data sources for many of the diseases listed in Table 1 are not available in Ireland, data from the national transplant services (cardiopulmonary, liver and renal diseases) has been linked to cancer registry data ^60^ and could be used to identify cohorts of patients with palliative care needs.

## Conclusions

Health and social care data collections are a powerful tool for PEoLC research ^1, 2, 24^ and these are available in Ireland ^16– 18^. Previous studies have shown that, with the appropriate permissions, detailed knowledge of the datasets and good study design, these data can be used for PEoLC research in Ireland ^36, 37^. Since 2018, more stringent requirements around data governance, data sharing and the requirement for informed consent arising from legislative changes to GDPR and Irish Health Research Regulations have impacted on the use of administrative health and social care data for research. The planned reforms of the Irish health services ^17, 67^ together with the HIQA recommendations for standards for data quality ^30^ should improve the Irish health information infrastructure and research potential of administrative health and social care data. Streamlining the existing fragmented health service should clarify data governance and ownership issues. Improved data standards requiring data models, data dictionaries and the development of minimum datasets will allow researchers to evaluate the research potential of a dataset in advance and gauge the level of effort required to access and use the data. The introduction of IHI’s for both service users and providers will improve data privacy by negating the need to store identifiable data name and/or date of birth etc. more than once. The increased security provided by IHI’s will facilitate data pseudonymisation while data linkage and data sharing based on a common IHI between datasets, i.e. deterministic linkage rather than probabilistic matching should be possible. The introduction of EHRs will transform data sharing across health care settings and IHIs are a key enabler of this. These changes will take time to fully implement but should allow the full power of administrative health and social care data for PEoLC research to be realised in due course.

## Data availability

### Underlying data

All data underlying the results are available as part of the article and no additional source data are required.
