# Supplementary material for: Using administrative health data for palliative and end of life care research in Ireland: potential and challenges
Source: HRB Open Res. 2021 May 26;4:17. Originally published 2021 Feb 9. [Version 2] doi: 10.12688/hrbopenres.13215.2 (PMC8014706; doi:10.12688/hrbopenres.13215.2)
Supplement: Peer review history

# Peer review: Reviewer response for version 1

**DOI:** 10.21956/hrbopenres.14367.r29182

**Published:** 2021-03-31

**Authors:** Kathleene Bennett

**Affiliations:** 1 Division of Population Health Sciences, Royal College of Surgeons in Ireland, Dublin, Ireland

This article provides a good review of the different datasets available in Ireland for research in palliative and end of life care research and is well presented overall. Only minor comments or suggestions are provided.

 In relation to the hospital inpatient enquiry (HIPE) data it may be worth including information on the validation of palliative care data when referring to initially, as it is mentioned later in the article.

 The Primary Care Reimbursement services (PCRS) is part of the HSE but this is not clear from the description. Also, the PCRS is involved in the administration of pharmacy claims data, not primary care prescription data as referred to in the article. For example, ‘Cancer registry data has also been linked to primary care prescription data to examine…’. Please amend accordingly.

 In the description of TILDA and confirmed deaths – up to which wave of the TILDA cohort was the information on confirmed deaths and matched death records provided for? Is there a reference to a report or other publication for this?

 The order of the datasets presented in the ‘results’ section could be consistent with the initial description in the first paragraph. For example, ‘These include population based death certificate data, hospital based episode data ….four disease registry collections and one nationally representative cohort study ..’ If the datasets are described in this sequence, the Cancer Registry data should perhaps be described with other registry data sets and not first.

 There have been recent changes (Jan 2021) to the Health Research Regulations in Ireland which make some of the statements on this are now out of date. Can the authors include these most recent changes? https://www.gov.ie/en/publication/b46c2-amendments-to-health-research-regulations/.

 There are examples of the use of electronic health records for PEoLC research in some countries and linkages to these e.g. UK that might be worth mentioning. Also electronic health records are used in some settings in Ireland.

 The conclusions appear to be focused on administrative data, however, not all registries or datasets are based on administrative data. Also, the conclusions include ‘We have shown that, with the appropriate permissions, detailed knowledge of the datasets and good study design,…’ but this review does not refer to study design in any detail. Therefore, some amendment to the conclusion (and the abstract) is required.

Is the work clearly and accurately presented and does it cite the current literature?

Yes

If applicable, is the statistical analysis and its interpretation appropriate?

Not applicable

Are all the source data underlying the results available to ensure full reproducibility?

Yes

Is the study design appropriate and is the work technically sound?

Yes

Are the conclusions drawn adequately supported by the results?

Partly

Are sufficient details of methods and analysis provided to allow replication by others?

Yes

Reviewer Expertise:

Epidemiology and biostatistics

I confirm that I have read this submission and believe that I have an appropriate level of expertise to confirm that it is of an acceptable scientific standard.

# Response

**Published:** 2021-05-13

**Authors:** Ailish Hannigan

**Affiliations:** University of Limerick, Ireland

Thank you Professor Bennett for reviewing our research paper and for your constructive feedback and comments. Based on these, we have substantially re-organised the layout of our manuscript and revised some of the content. Please see below for a detailed point by point response to each comment (reviewer's comments in bold).

**Comment 1: In relation to the hospital inpatient enquiry (HIPE) data it may be worth including information on the validation of palliative care data when referring to initially, as it is mentioned later in the article.**

 Response: We have moved the paragraph ‘Opportunities for data validation’ to the end of the paragraph describing hospital episode data.

**Comment 2: The Primary Care Reimbursement services (PCRS) is part of the HSE but this is not clear from the description.**

 Response: This has been clarified in the paragraph ’Primary Care Reimbursement Service data' in the first sentence.

**Comment 3: Also, the PCRS is involved in the administration of pharmacy claims data, not primary care prescription data as referred to in the article. For example, ‘Cancer registry data has also been linked to primary care prescription data to examine…’. Please amend accordingly.**

 Response: This error has now been corrected. We have renamed the paragraph ‘Primary Care prescription data’ to ‘Primary Care Reimbursement Service data’ and amended the sentence ‘Cancer registry data has also been linked to primary care prescription data to examine…’ to read 'Cancer registry data has also been linked to PCRS pharmacy claims data to examine…’. We have also replaced the phrase ‘community based prescription data’ with the term pharmacy claims data in the abstract and main text.

**Comment 4: In the description of TILDA and confirmed deaths – up to which wave of the TILDA cohort was the information on confirmed deaths and matched death records provided for? Is there a reference to a report or other publication for this?**

 Response: Matching was performed for all individuals who died between Wave 1 (2009/2011) and March 2018. We have restructured the paragraph to include this information and clarify the reference for the published paper.

**Comment 5: The order of the datasets presented in the ‘results’ section could be consistent with the initial description in the first paragraph. For example, ‘These include population based death certificate data, hospital based episode data ….four disease registry collections and one nationally representative cohort study ..’ If the datasets are described in this sequence, the Cancer Registry data should perhaps be described with other registry data sets and not first.**

 Response: We have re-organised the layout so that the datasets are now ordered as suggested, that is death certificate data, hospital based episode data, primary care reimbursement service data, the Irish Longitudinal Study on Ageing, cancer registry data and other disease registries. We have also rearranged table 2 to reflect this order. This change has also been made in the Abstract.

**Comment 6: There have been recent changes (Jan 2021) to the Health Research Regulations in Ireland which make some of the statements on this are now out of date. Can the authors include these most recent changes? https://www.gov.ie/en/publication/b46c2-amendments-to-health-research-regulations/.**

 Response: We have amended the second paragraph, section ‘General Data Protection Regulations’ to outline the main changes arising from the recent amendments to the Health Research Regulations.

**Comment 7: There are examples of the use of electronic health records for PEoLC research in some countries and linkages to these e.g. UK that might be worth mentioning. Also electronic health records are used in some settings in Ireland.**

 Response: We have added a subheading ‘Electronic Health Records’ under the section ‘Considerations for PEoLC research’. We describe the progress to-date on the introduction of a national Electronic Health Record in Ireland. We also describe how they have been used in Scotland and England for PEoLC research.

**Comment 8: The conclusions appear to be focused on administrative data, however, not all registries or datasets are based on administrative data.**

 Response: We have broadened the first sentences of the conclusions to be inclusive of all health and social care data collections.

**Comment 9: Also, the conclusions include ‘We have shown that, with the appropriate permissions, detailed knowledge of the datasets and good study design,…’ but this review does not refer to study design in any detail. Therefore, some amendment to the conclusion (and the abstract) is required.**

 Response: We have amended the abstract and the paper to remove any sentences that imply we have described study design in this review.

# Peer review: Reviewer response for version 1

**DOI:** 10.21956/hrbopenres.14367.r28879

**Published:** 2021-03-08

**Authors:** Wei Gao

**Affiliations:** 1 Florence Nightingale Faculty of Nursing Midwifery and Palliative Care, Cicely Saunders Institute, King’s College London, London, UK

This is a well-written article, providing a good overview of the datasets available for PEoLC research. I have only some minor comments/suggestions as below. 

 Title: should the “linked” be removed, as the identified eight datasets can be used with or without linkage for PEoLC research? I can also see the “linked” has been scattered throughout the manuscript, I feel the adjective unnecessarily narrows down the potential use of the data. I would also encourage you to consider adding a “social” aspect to the datasets, such as “health and social care datasets”.  

 Primary care prescription data. Should this be “primary care reimbursement data”, or the information contained in the data set is primarily prescribing data? i.e. is there any care data/reasons for prescriptions recorded.     

   

 “Where people die and their preference for place of death are important indicators of the quality of end-of-life care.” Although at the population level, place of death may be a quality indicator for end-of-life care, place of death at the individual-level does not necessarily indicate the care quality. See this paper (Pollock K. BMJ. 2015 Oct 7;351:h4855 ^1^). Please revise. 

 It is worth mentioning the rough costs associated with accessing the potential datasets for research. Or is it free in most of the cases? And also, it would be good to highlight in the Discussion that the data sources for most conditions with PEoLC need (i.e. COPD, renal, those listed in your Table 1) are lacking.

Is the work clearly and accurately presented and does it cite the current literature?

Yes

If applicable, is the statistical analysis and its interpretation appropriate?

Not applicable

Are all the source data underlying the results available to ensure full reproducibility?

Yes

Is the study design appropriate and is the work technically sound?

Yes

Are the conclusions drawn adequately supported by the results?

Yes

Are sufficient details of methods and analysis provided to allow replication by others?

Yes

Reviewer Expertise:

Big data; Routine data; Real-world evidence; Palliative and End of Life Care; Artificial Intelligence.

I confirm that I have read this submission and believe that I have an appropriate level of expertise to confirm that it is of an acceptable scientific standard.

## References

[1] : Is home always the best and preferred place of death?. *BMJ*.2015;351: 10.1136/bmj.h4855 h4855 10.1136/bmj.h4855 26446163

# Response

**Published:** 2021-05-13

**Authors:** Ailish Hannigan

**Affiliations:** University of Limerick, Ireland

Thank you Professor Gao for reviewing our research paper and for the constructive feedback given. We have revised our manuscript accordingly. Please see below a detailed point by point response to each comment (reviewer's comments in bold).

**Comment 1: Title: should the “linked” be removed, as the identified eight datasets can be used with or without linkage for PEoLC research? I can also see the “linked” has been scattered throughout the manuscript, I feel the adjective unnecessarily narrows down the potential use of the data.**

 Response: We agree the datasets can be used for PEoLC research without linkage and have removed the word linked from the title. Within the manuscript we have kept the word linked where different datasets have been linked or where we are describing our experience of using linked data.

**Comment 2: I would also encourage you to consider adding a “social” aspect to the datasets, such as “health and social care datasets”.**

 Response: We have addressed this issue as follows:

 1. We have amended the first line of the main text Introduction 

 2. We have replaced the term ‘administrative health data’ with ‘administrative health and social care data’ throughout the manuscript when referring to Irish health data. 

**Comment 3: Primary care prescription data. Should this be “primary care reimbursement data”, or the information contained in the data set is primarily prescribing data? i.e. is there any care data/reasons for prescriptions recorded.**

 Response: This is an error, we have renamed the paragraph to ‘Primary Care Reimbursement Service data’. We have also amended the sentence ‘Cancer registry data has also been linked to primary care prescription data to examine…’ to read 'Cancer registry data has also been linked to PCRS pharmacy claims data to examine…’. No care data are recorded by PCRS.

**Comment 4: “Where people die and their preference for place of death are important indicators of the quality of end-of-life care. “Although at the population level, place of death may be a quality indicator for end-of-life care, place of death at the individual-level does not necessarily indicate the care quality. See this paper (Pollock K. BMJ. 2015 Oct 7;351:h48551). Please revise.**

 Response: We agree, this sentence has been amended to read ‘Place of death is an important outcome measure at a population level.’

**Comment 5: It is worth mentioning the rough costs associated with accessing the potential datasets for research. Or is it free in most of the cases?**

 Response: To our knowledge there is no requirement for payment (for non-commercial organisations) to access any of the data sources mentioned here. We have added a sentence on this to the paper.

**Comment 6: And also, it would be good to highlight in the Discussion that the data sources for most conditions with PEoLC need (i.e. COPD, renal, those listed in your Table 1) are lacking.**

 Response: We agree. On further consideration of this point we have now included the national renal transplant registry to Table 2 and noted the existence of a number of other transplant services in Ireland [see Results section under the heading ‘National Renal Transplant Registry’]. In the final paragraph of the Discussion we acknowledge that data sources for most of the conditions listed in Table 1 are lacking and identify the national transplant services as potential data sources.
